# Establishment of an oral burn model in streptozotocin-induced diabetic rats

**DOI:** 10.1186/s40902-024-00453-6

**Published:** 2024-12-30

**Authors:** Su-Young Kim, Seong-Gon Kim, Dae-Won Kim, Ji-Hyeon Oh

**Affiliations:** 1 Department of Oral and Maxillofacial Surgery, College of Dentistry, Gangneung-Wonju National University, 7, Jukheon-Gil, Gangneung, Gangwondo 28644 Republic of Korea; 2 Department of Oral Biochemistry, College of Dentistry, Gangneung-Wonju National University, 7, Jukheon-Gil, Gangneung, Gangwondo 28644 Republic of Korea

**Keywords:** Burns, Oral ulcer, Diabetes mellitus, Wound healing, Rat

## Abstract

**Background:**

Oral ulcers are painful mucosal lesions prone to infection and inflammation. To evaluate the effectiveness of treatments, a suitable experimental animal model with an appropriate healing period is required. The aim of this study was to develop an animal model for oral ulcer research by comparing oral burn wounds of different sizes and locations in diabetic rats.

**Methods:**

Forty-four male Sprague–Dawley rats with induced diabetes were divided into six groups based on burn wound location and size: T5 (*n* = 10, tongue 5 mm), T3 (*n* = 10, tongue 3 mm), P5 (*n* = 10, palate 5 mm), P3 (*n* = 10, palate 3 mm), CT (*n* = 2, control tongue), and CP (*n* = 2, control palate). The burn wounds were induced by applying a heated device (100–120 °C) for 3 s. At 1- and 2-weeks post-surgery, macroscopic examination, histological staining, immunohistochemistry, and Western blot analysis were performed to compare the healing progress.

**Results:**

Healing progressed more rapidly in the second week than in the first for all groups, with burns on the tongue (Groups T5 and T3) showing more advanced healing compared to burns on the palate (Groups P5 and P3). By the second week, Group T3 was almost completely healed, while Group T5 had some remaining wounds. In contrast, Groups P5 and P3 showed minimal healing. This faster healing on the tongue was further supported by significantly lower expression levels of TNF-α and IL-1β and a reduction in ulcer size, particularly on the tongue compared to the palate.

**Conclusion:**

A 3 mm or 5 mm burn wound on the tongue of diabetic rats can serve as a useful animal model for evaluating new treatments for wound healing, particularly up to the first week. However, for studies extending to the second week, the 5 mm burn wound model on the tongue might be more advantageous.

**Supplementary Information:**

The online version contains supplementary material available at 10.1186/s40902-024-00453-6.

## Background

Oral ulcers, characterized by open sores inside the mouth resulting from a break in the mucous membrane or epithelium, are the most common painful mucosal lesions [1–3]. They typically exhibit periodic onset of burning pain and are highly susceptible to infection, often leading to inflammation and tissue necrosis. They can range from mild to severe pain, often caused by a variety of factors such as trauma, infection, medical conditions, medications, or other nonspecific processes [1, 4]. The most frequent type of oral mucosa ulcer is aphthous ulcer, which is a benign, self-limited condition that typically heals on its own within two weeks [5, 6]. Despite their transient nature, aphthous ulcers can be quite uncomfortable for those who experience them, and they can make eating, drinking, and speaking difficult.

To reduce the risk of infection and discomfort for patients, various treatment methods have been developed and evaluated to alleviate symptoms and promote rapid wound healing of oral soft tissue [2, 3, 7–13]. Some of these treatments include topical corticosteroids, oral analgesics, antimicrobial agents, and dietary supplements [5, 14]. The choice of treatment will depend on the severity of the ulcers, the underlying cause of the ulcers, and the individual’s overall health status.

To create novel treatment methods, it is necessary to establish an evaluation model for new reagents [8]. Although rats and mice are often used as lab animals, their small oral cavity limits the size of experimental ulcers. As a result, the time required for healing is often too short to accurately assess the efficacy of tested reagents. For a reliable evaluation of new reagents, it is essential that natural healing does not occur too quickly or fail altogether, and that the experimental animals do not suffer significant mortality due to the wounds. In other words, an animal model is needed that exhibits an appropriate healing period and does not cause severe pain and stress leading to death in the experimental animals.

Compared to healthy rats, diabetic rats have delayed wound healing [12, 15–17]. Additionally, burn wounds tend to heal more slowly than traumatic wounds [18]. Consequently, diabetic rats with oral burn wounds can serve as a suitable experimental animal model with an appropriate healing period to evaluate the healing effects of new reagents for the treatment of oral ulcers, as the wounds heal relatively slowly, making it easier to compare and assess the effectiveness of new treatments. However, ulcers of 4 mm in size on the buccal mucosa of diabetic rats are associated with high mortality rates [19], underscoring the necessity for a burn model that offers higher survival rates.

Therefore, the purpose of this study was to establish a more viable animal model for oral ulcer research by comparing oral burns of different sizes and regions in diabetic rats. To achieve this, burns were induced to create tongue or palate ulcers of two sizes in diabetic rats. Each type of ulcer was then compared by evaluating the wound healing progression and the expression levels of inflammation-related cytokines at one- and two-weeks post-injury.

## Material and methods

### Animals

The animal study was approved by the Institutional Animal Care and Use Committee at Gangneung-Wonju National University (GWNU-2022–6). Forty-four male Sprague–Dawley rats aged 7 weeks with a body weight range of 210 to 240 g were obtained from Samtako Bio Inc (Osan, Republic of Korea). The sample size for this study was determined using the G*Power software 3.1.9.7 (Heinrich-Heine-Universität Düsseldorf, Germany). The calculation was based on an effect size of 0.5, an α error probability of 0.05, and a power (1-β) of 0.7. Prior to the experiment, the rats were allowed to acclimate to the conditions for 1 week, with two rats housed together in each cage. The animals were kept in a controlled environment with a 12-h light and 12-h darkness cycle at a temperature of 20 to 22 ℃ and 40% humidity.

### Experimental design

For the STZ injection, the rats' body weight ranged from 240–280 g, and they fasted for 6–8 h with access to water. To induce type 1 diabetes, a single intravenous injection of streptozotocin (STZ) (50 mg/kg) dissolved in citrate-buffered saline solution (pH 4.5) was administered to the rats under inhalation anesthesia through the tail vein. Subsequently, the rats were placed back in their cages and provided with standard food and 10% sucrose water. After 3 days, their blood glucose levels were measured from the tail vein under 6–8 h fasting conditions. Rats with blood glucose levels above 300 mg/dL were selected for further investigation, while rats with inadequate blood glucose levels were given an additional STZ injection.

**Fig. 1:**
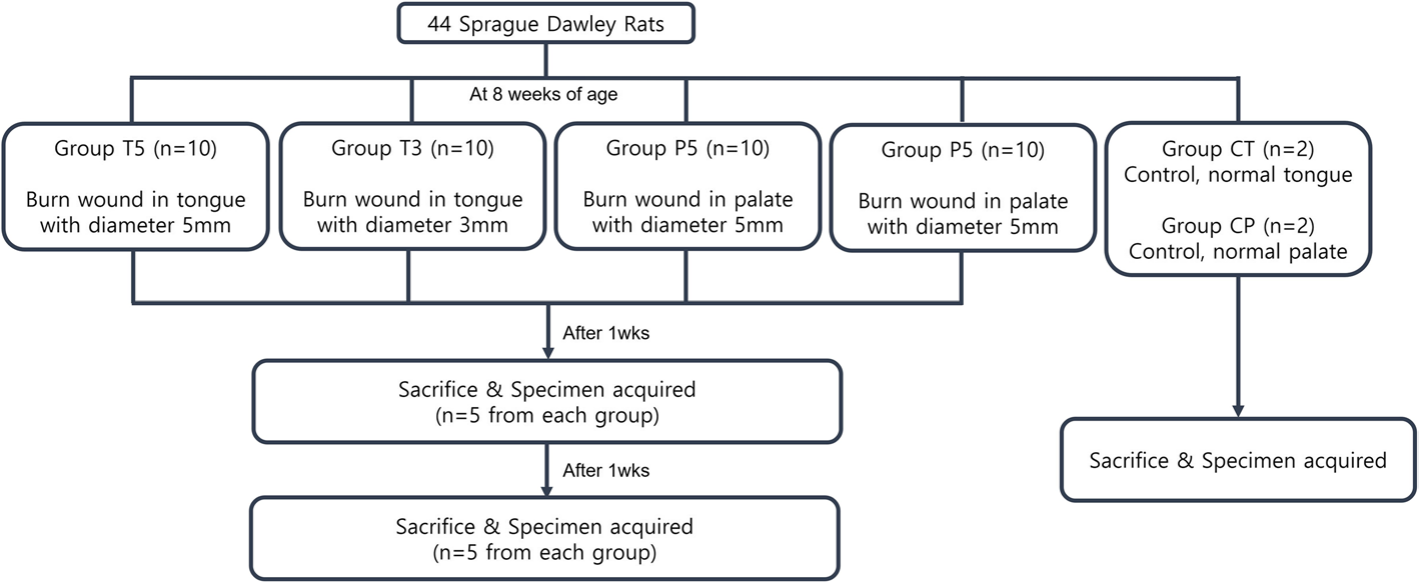
Schematic diagram of the study design and animal grouping

STZ-injected rats were randomly assigned to six groups based on burn wound location and size as follows: Group T5 (*n* = 10, tongue with diameter 5 mm and depth 1 mm); Group T3 (*n* = 10, tongue with diameter 3 mm and depth 1 mm); Group P5 (*n* = 10, palate with diameter 5 mm and depth 1 mm); Group P3 (*n* = 10, palate with diameter 3 mm and depth 1 mm); Group CT (*n* = 2, control normal tongue); Group CP (*n* = 2, control normal palate). Figure 1 provides a concise summary of the animal study. To minimize potential confounders, rats within each cage were the same group, but the cages were arranged so that cages of different groups were placed next to each other, ensuring that the cage location did not influence the study outcomes. Group allocation was known only to the corresponding author, while all other researchers involved in the study were blinded to the group allocations to ensure unbiased conduct and analysis.

**Fig. 2:**
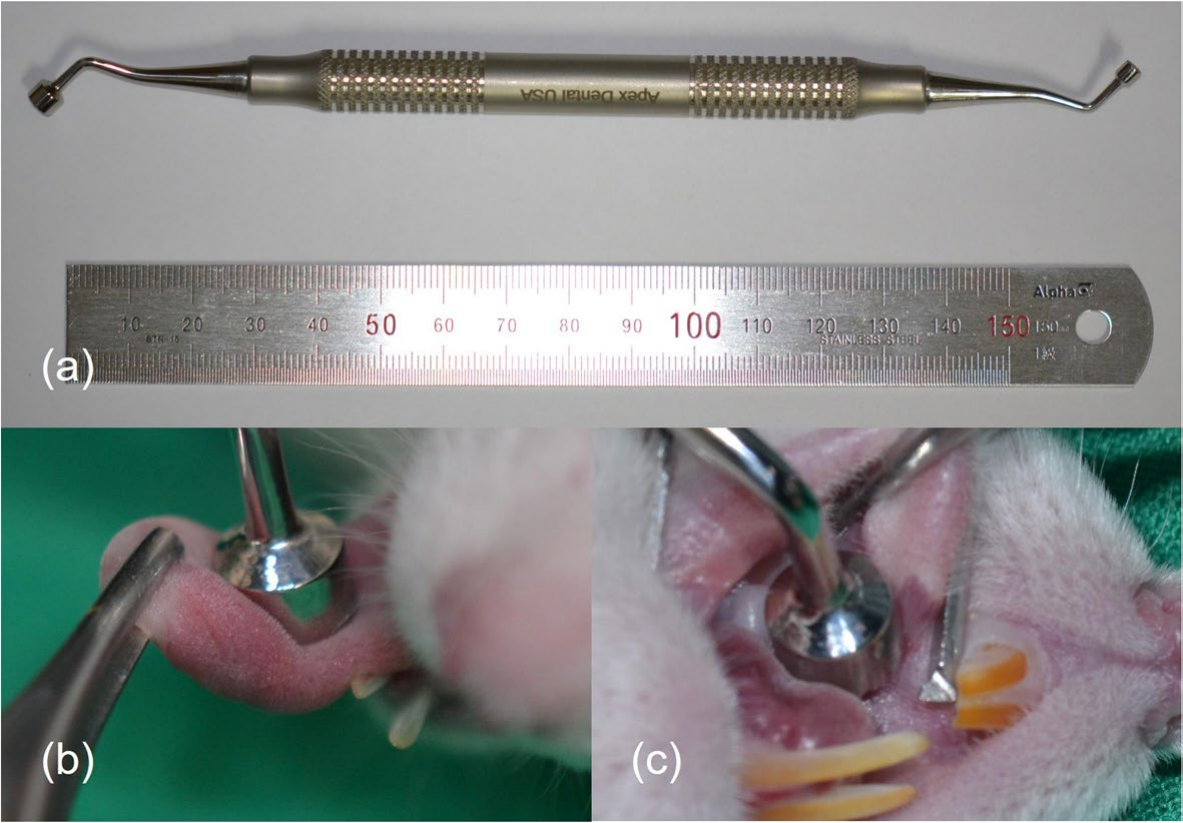
**a** Device used for inducing burn wounds. **b** Application of the device to the tongue. **c** Application of the device to the palate

Anesthesia was administered to the rats through intramuscular injection of Zoletil (Virbac Korea, Seoul, Korea) and Rompun (Bayer Korea, Seoul, Korea) before creating the burn wounds. The burn wounds were induced by heating a device (Fig. 2) to 100–120 °C and then applying it to the tongue or palate for 3 s. Additionally, each rat received intramuscular injections of gentamicin (5 mg/kg; Samu Median, Seoul, Republic of Korea) and tolfenamic acid (0.1 mL/kg; Samyang Anipharm, Seoul, Republic of Korea) form immediately after the operation until the second day post-operation to facilitate the healing process.

To assess the healing progress of the burn wounds four rats from each experimental group were sacrificed at 7 days and six rats at 14 days after the creation of the burn wound by administering an overdose of the anesthetic Zoletil via intraperitoneal injection. After a clinical assessment of wound healing progression, the wound specimens were collected and cut in half; one half was used for histological analysis, while the other half was utilized for Western blot analysis.

### Histological staining and immunohistochemistry

Full-thickness biopsies were obtained from the tongue or palate mucosa and preserved in 4% paraformaldehyde. To conduct hematoxylin and eosin (H&E) staining, 5 μm sections of the formalin-fixed paraffin-embedded tissue samples were deparaffinized using xylene to eliminate the paraffin wax. The sections were then gradually rehydrated by passing them through a series of alcohol solutions. Subsequently, the sections were immersed in a hematoxylin solution for 6 min, followed by rinsing with distilled water. To remove excess hematoxylin and differentiate the nuclei, the sections were treated with an acid-alcohol solution for a specific duration. Counterstaining with eosin was performed, and after the dehydration process, the sections were mounted with a coverslip.

For immunohistochemical staining of tumor necrosis factor-alpha (TNF-α) (Abcam, Cambridge, UK) and interleukin-1 beta (IL-1β) (Santa Cruz Biotechnology, Santa Cruz, CA, USA), the rehydration process followed the protocol for H&E staining. After blocking the activity of endogenous peroxidase using 30% H2O2 (Samchun, Pyeongtaek, Republic of Korea) for 7 min, the slide underwent two washes with phosphate-buffered saline (PBS). Subsequently, protein block (CAT#: X0909, Agilent Technologies, Santa Clara, CA, USA) was applied for 1 h to prevent nonspecific binding. Primary antibodies (dilution ratio 1:100) were then applied to each section and left to incubate overnight at 4 °C. The slide was later washed three times with PBS, followed by application of a commercially prepared horseradish peroxidase-conjugated secondary antibody (Real EnVision™, Dako, Glostrup, Denmark) for 30 min. After an additional three washes with PBS, chromogen (DAB + chromogen, Dako) was applied for 5 min. The sections were then rinsed with distilled water and covered with a mounting medium (Ultramount Aqueous Permanent Mounting Medium, Dako) before placing a coverslip. The histological images were captured using a digital camera (DP-72, Olympus, Tokyo, Japan).

### Western blot

After determining the protein concentration, Western blotting was performed to analyze TNF-α and IL-1β using primary antibodies from Santa Cruz Biotechnology (Santa Cruz, CA, USA). Tissue samples were homogenized in a lysis buffer containing protease inhibitors and centrifuged at 10,000 × g for 10 min at 4 °C to remove cellular debris. The supernatant's protein concentration was assessed, followed by mixing the protein samples with SDS sample buffer and heating at 95–100 °C for 5 min. Equal amounts of protein were loaded onto an SDS-PAGE gel and electrophoresed using a Tris–glycine buffer system. The separated proteins were then transferred onto a PVDF membrane using a transfer apparatus. For Western blot analysis, the PVDF membrane was blocked and subsequently incubated overnight at 4 °C with primary antibodies against TNF-α and IL-1β diluted in blocking buffer (dilution ratio = 1:1000). Following primary antibody incubation, the membrane was washed and incubated with a secondary antibody conjugated to a detection enzyme for 1 h at room temperature. After washing, the membrane was developed using a chemiluminescent substrate, and the protein bands were visualized using a chemiluminescence imaging system. All experiments were conducted in triplicate to ensure consistency and reliability of results. ImageJ (National Institutes of Health, Bethesda, MD, USA) was utilized to analyze the expression levels of each protein in the gel photo by comparing them to the expression level of β-actin.

### Statistical analysis

All statistical analyses were performed using SPSS 28 software (SPSS Inc., Chicago, IL, USA). The protein expression differences in tissue samples were evaluated using one-way analysis of variance (ANOVA). Subsequently, the Bonferroni method was applied as the post-hoc test. A *p*-value < 0.05 was considered statistically significant.

## Results

### Wound healing progression

**Fig. 3:**
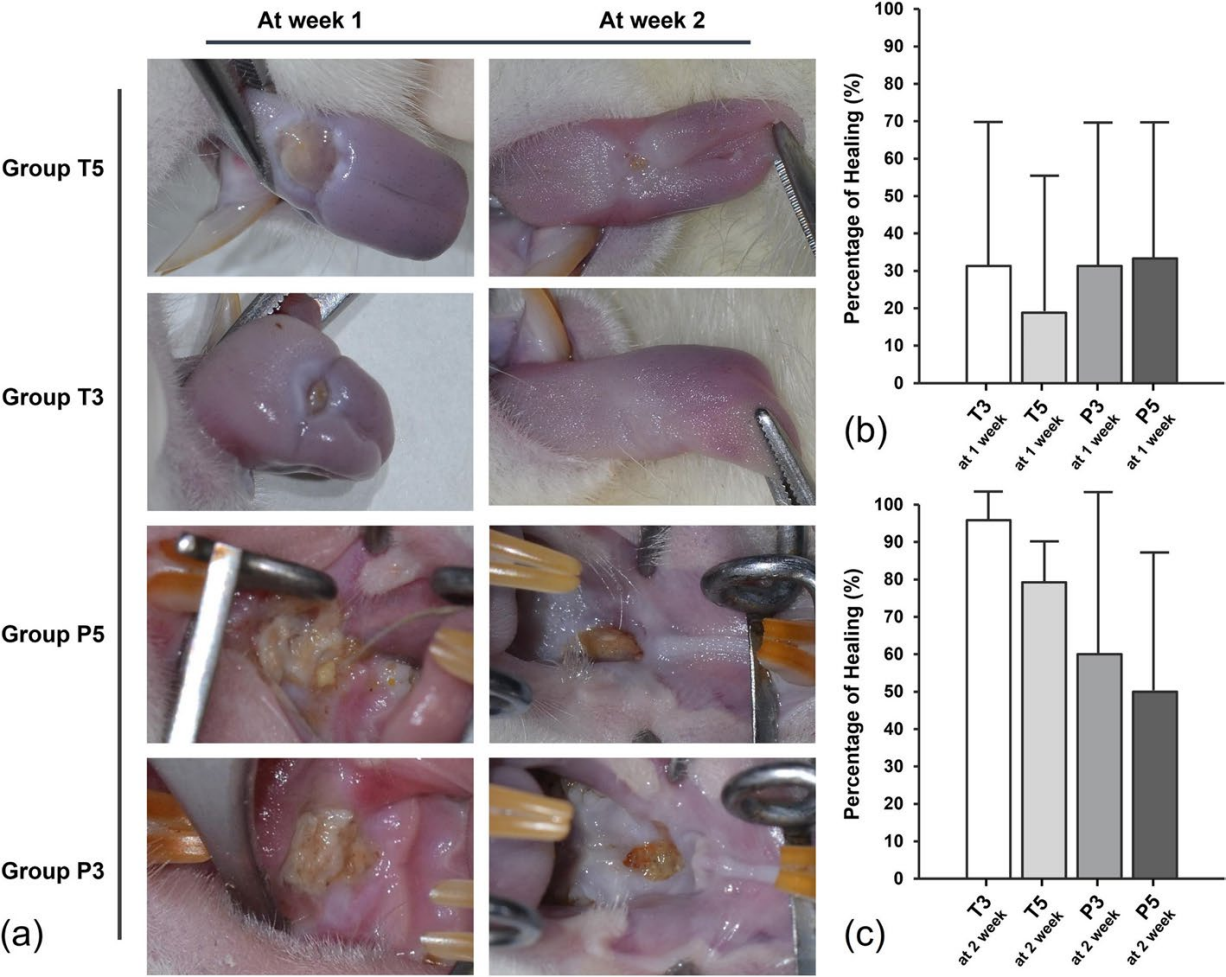
**a** Healing progression of burn wounds over time in each group. Group T3 exhibited the fastest healing, with almost complete wound closure by the second week. Group T5 also demonstrated advanced healing, although some wounds remained by the second week. Groups P3 and P5 showed significantly slower healing, with minimal improvement by the second week. **b** Percentage of Healing at 1 Week Post-Injury in Diabetic Rats. Healing was observed in all groups, with the groups T3 and T5 showing higher healing percentages compared to the groups P3 and P5. **c** Percentage of Healing at 2 Week Post-Injury in Diabetic Rats. At 2 weeks, healing progressed, with Group T3 showing nearly complete healing. Group T5 also exhibited substantial healing, whereas the groups P3 and P5 showed slower progression

Figure 3 shows the healing patterns of burn wounds over time in each group. Groups T5 and T3, which received burns on the tongue, exhibited more advanced healing compared to Groups P5 and P3, which had burns on the palate. By the second week, Group T3 almost completely healed, while T5 still showed some remaining wounds. On the other hand, Groups P5 and P3 showed minimal healing even by the second week, with some cases displaying exposed palatal bone. Although there was a slight reduction in palatal bone exposure from day 7 to day 14 in certain rats, there were still cases where bone exposure persisted even after 2 weeks.

### Histological and immunohistochemical analysis

**Fig. 4:**
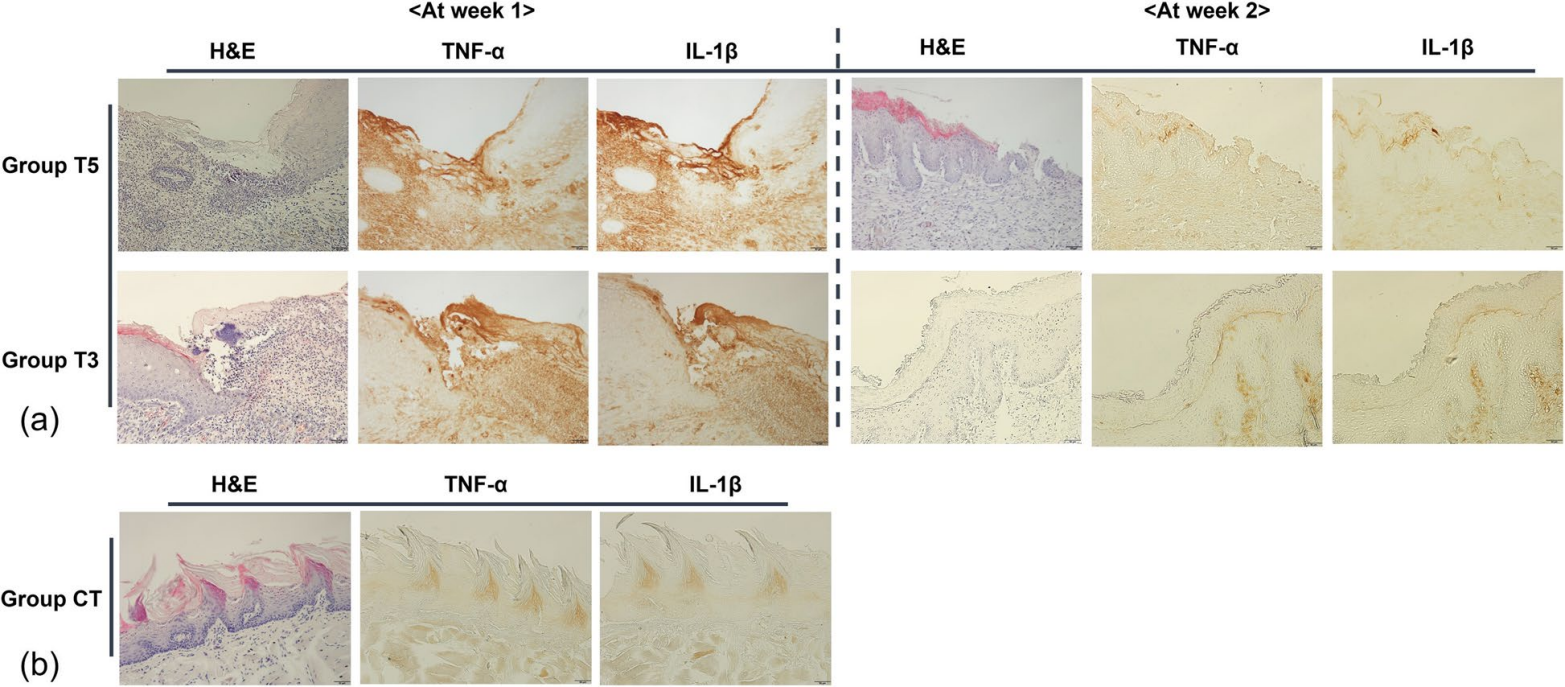
**a** Epithelial continuity changes and TNF-α, IL-1β immunostaining in T5 and T3 groups over 2 weeks. Epithelial continuity gradually improved in Group T5 but was not fully restored by the second week, whereas Group T3 showed complete epithelial restoration within the same timeframe. Immunohistochemical analysis revealed elevated TNF-α and IL-1β expression in both groups at week 1, with Group T5 maintaining slightly higher levels at week 2, while Group T3's expression levels returned to near-control values. **b** Immunostaining results for the control group (Group CT) are shown for comparison

In Fig. 4a, the changes in epithelial continuity and the results of TNF-α and IL-1β immunostaining in Groups T5 and T3 with burns on the tongue over 1 and 2 weeks of healing are presented. Group T5 showed a more improved epithelial continuity by the second week, although not fully restored, while Group T3 exhibited complete restoration by the second week. The immunostaining for TNF-α and IL-1β showed higher expression levels in groups T5 and T3 compared to the control group (Group CT, Fig. 4b) at week 1, but by week 2, group T5 had slightly higher expression, while the expression of group T3 was similar to the control.

**Fig. 5:**
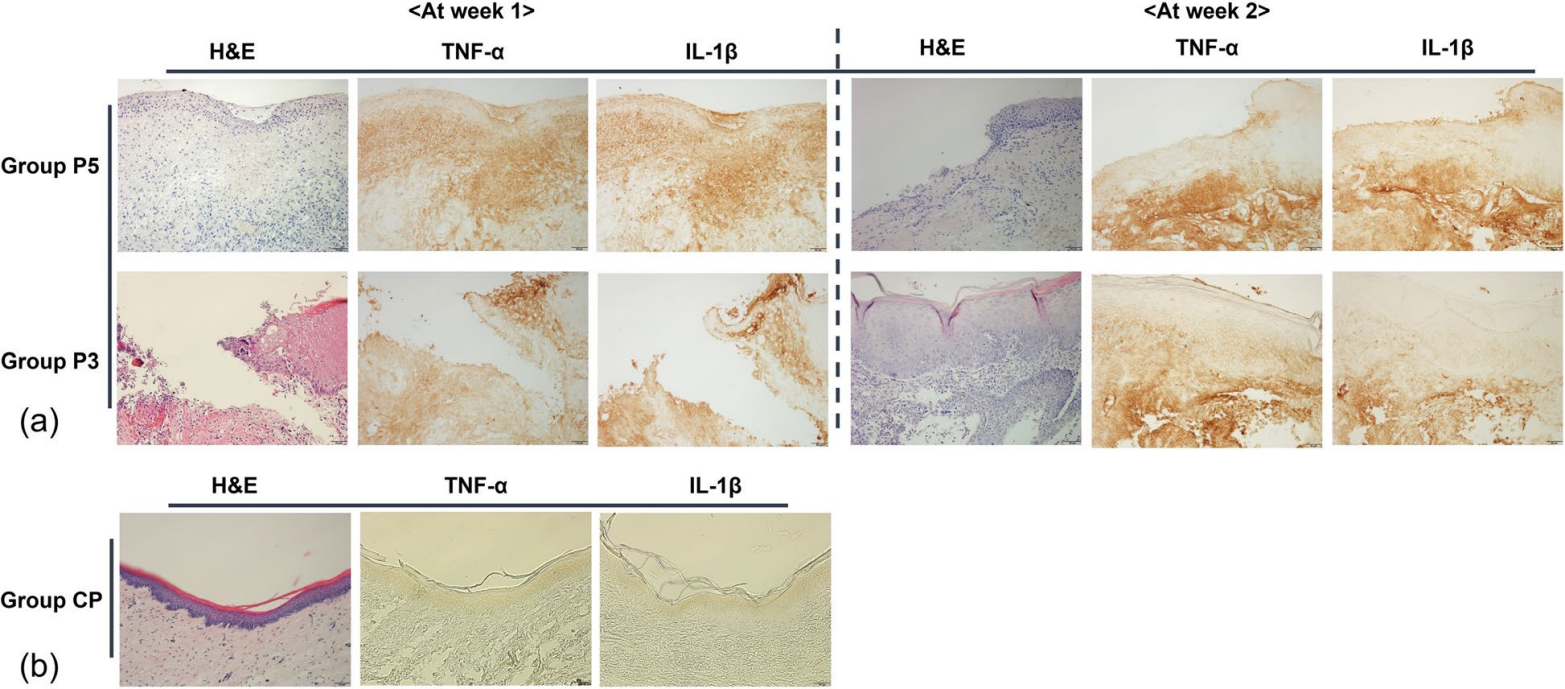
**a** Epithelial continuity changes and TNF-α, IL-1β immunostaining in P5 and P3 groups over 2 weeks. Group P5 exhibited persistent disruption in epithelial continuity by the second week, with no significant healing progress, while Group P3 showed partial recovery. TNF-α and IL-1β expression remained elevated in both groups throughout the 2-week period, with little change from the initial levels observed at week 1. **b** Immunostaining results for the control group (Group CP) are presented for comparison

Figure 5a displays the changes in epithelial continuity and the results of TNF-α and IL-1β immunostaining in Groups P5 and P3 with burns on the palate over 1 and 2 weeks of healing. Group P5 appeared to show no improvement in epithelial continuity by the second week, while P3 exhibited partial restoration by the same period. The immunostaining for TNF-α and IL-1β showed higher expression levels in groups P5 and P3 compared to the control group (Group CP, Fig. 5b) at week 1. The expression levels in groups P5 and P3 remained similar at week 2 to those observed at week 1.

### Western blot analysis

**Fig. 6:**
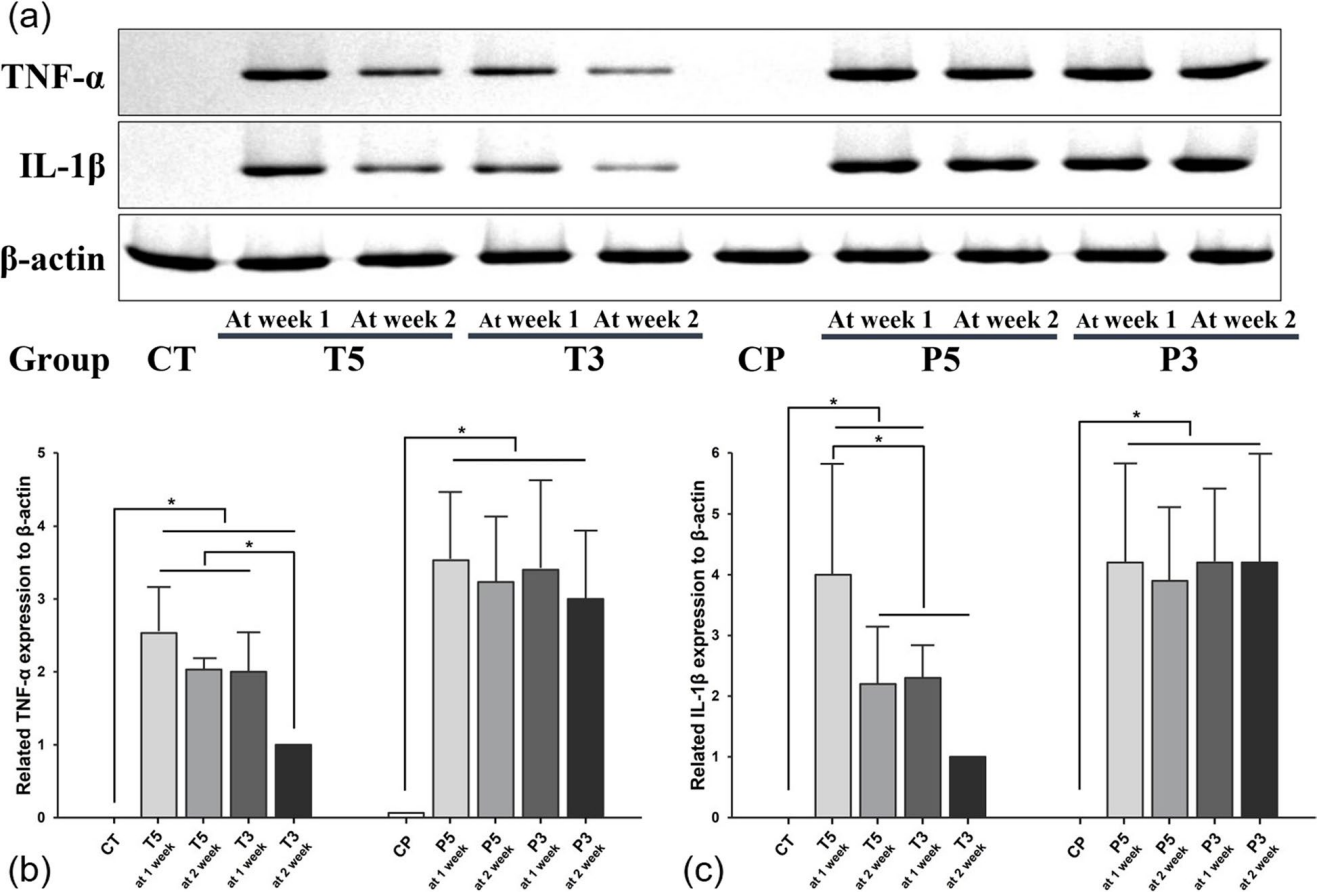
**a** Western blot analysis of TNF-α and IL-1β expression. **b** TNF-α expression levels in different groups over time. T5 and T3 exhibited significantly higher TNF-α levels at 1 week compared to the control (CT), with a slight decrease by week 2. Group T3 showed a statistically significant reduction in TNF-α expression by the second week, whereas Group T5 maintained relatively high levels. Groups P5 and P3 maintained consistently high TNF-α levels across both time points, with no significant changes. **c** IL-1β expression levels in different groups over time. A marked increase in IL-1β expression was seen in Group T5 at 1 week, which significantly decreased by the second week. Group T3 also demonstrated a reduction in IL-1β levels by week 2, approaching the control group (CT) levels. Groups P5 and P3 showed persistently high IL-1β expression with no significant reduction over time

The results of Western blot analysis for TNF-α and IL-1β were consistent with the immunostaining results (Fig. 6a). The results showed significant differences in inflammatory cytokine expression across different groups and time points. TNF-α expression was statistically significantly higher in Groups T5 and T3 at week 1 compared to the control group (CT). This increased expression persisted until week 2, but both groups showed a slight decrease, with only Group T3 showing a statistically significant reduction (*p* < 0.05) (Fig. 6b). A similar trend was observed for IL-1β expression. As shown in Fig. 6c, Group T5 exhibited a significant increase in IL-1β levels at week 1, followed by a statistically significant decrease at week 2 (*p* < 0.05). Group T3 showed a decrease in IL-1β expression at week 2 compared to week 1, approaching the levels seen in the control group.

In contrast, Groups P5 and P3 exhibited consistently high levels of both TNF-α and IL-1β throughout the 2-week period. As shown in Fig. 6b and c, there was no significant change in expression levels between the first and second weeks for either cytokine, indicating a sustained inflammatory response in these groups. Both Groups P5 and P3 had significantly higher TNF-α and IL-1β expression compared to the control group (CP) at both time points, with no observable trend towards reduction (*p* < 0.05).

## Discussion

Selecting a model with the appropriate ulcer size and location is crucial for accurately assessing new treatment strategies for oral ulcers. This study demonstrated that diabetic rats with oral burn wounds could be effectively used as an animal model for oral ulcer research. The delayed wound healing observed in these rats provides a reliable framework for evaluating the efficacy of therapeutic agents designed to treat oral ulcers.

Various methods have been reported for creating oral wounds in rats, with most studies using blades or punches to induce traumatic ulcerations [4, 8–10]. The healing period varies depending on the location and size of the wound, but these excision wounds generally heal naturally within 7 to 14 days [15, 20]. Additionally, there have been studies that created chemical burn wounds in the oral cavity of rats using substances such as phenol and acetic acid [1–3]. One study reported that when phenol was used to create oral wounds in rats, only about 5% of the wounds healed naturally even after 18 days [1]. Chemical or thermal burn wounds tend to heal more slowly compared to traumatic wounds [18]. Animal models for burn wounds have primarily focused on the skin, with no studies reported on creating them in the oral mucosa [21]. The most frequently used animal for burn research is the rat, due to its availability, low cost, resistance to infection, and the ability to replicate various types of burns [22]. Typically, burn wounds have been created by directly applying heated metal or water to the shaved dorsum skin of rats [22–24]. When a stainless-steel rod with a diameter of 1 cm was heated to 100 °C and applied to the skin for 5 s, a full-thickness burn occurred, and contact for more than 10 s resulted in damage extending to the entire dermis, subcutaneous tissue, and parts of the underlying skeletal muscle [25]. The severity of the burn was related to the age of the rat and the duration of application; in two-month-old rats, a 1 cm diameter metal rod heated to 100 °C caused a superficial second-degree burn with 3 s of contact and a deep second-degree burn with 5 s of contact [24]. Although there are differences between skin and mucosa, our study also applied a stainless-steel instrument heated to 100–120 °C for 3 s on 8-week-old rats, and it is believed that this likely resulted in superficial to deep second-degree burns.

Various sites for creating oral wounds in rats have been reported. Generally, wounds are formed on the palate [8, 9], buccal mucosa [3, 8, 10, 15], tongue [4, 20], and the labial fornix region of the inferior incisors [2, 13]. However, no study has compared the healing of wounds based on the site where they are induced using the same methods. Therefore, this study aimed to establish an experimental animal model with an appropriate healing rate by creating burn-induced oral ulcers of two different sizes at two different sites. We then compared the healing progression over time and analyzed the expression levels of inflammation-associated cytokines TNF-α and IL-1β. The results showed that the expression levels of TNF-α and IL-1β were significantly higher in the group with delayed wound healing, suggesting an enhanced inflammatory response [26]. Excessive inflammation can delay tissue regeneration and impede wound healing [4]. Specifically, elevated TNF-α is associated with chronic and slow-healing acute wounds [27]. The overexpression of TNF-α and IL-1β increases the infiltration and activation of inflammatory cells, leading to a chronic inflammatory state. This process promotes cell apoptosis and accelerates the degradation of the extracellular matrix, acting as a factor that hinders tissue regeneration and recovery [28]. Thus, the observation of high TNF-α and IL-1β expression in the group with impaired wound healing suggests that these inflammatory cytokines were not adequately regulated at the site of tissue damage, preventing the normal progression of the healing process.

Oral tissues share common histological features such as surface epithelium and lamina propria, but the palate differs clinically and histologically from other oral tissues [29, 30]. Wounds on the tongue, which consists of mucosa covering loose connective tissue and muscle, heal more quickly than wounds on the palate, which is attached gingiva covering the alveolar bone, because epithelial coverage progresses rapidly and does not rely on stromal healing [31]. Similarly, in this study, it was observed that wounds on the tongue healed more completely than palatal wounds. Notably, in the 3 mm tongue wound group, complete healing was observed in some rats within just one week. If wounds heal too quickly naturally, the difference in results between the control group and the experimental group is minimal, making it difficult to compare the effectiveness of the new reagents. In contrast, delayed healing was observed in 5 mm tongue wounds after two weeks. Therefore, using a 5 mm tongue model may effectively demonstrate differences in healing patterns between the control group and experimental group, making it suitable for evaluating the effects of new treatment regimens.

Additionally, oral wound healing typically involves the healing of palatal and gingival tissues in the presence of healthy underlying bone and without scar tissue formation. Healing of palatal wounds is more challenging in the absence of healthy underlying bone [32]. A burn wound on the palate may affect the underlying bone due to the thin mucosa, and if thermal osteonecrosis occurs in the bone, the healing of the overlying soft tissue becomes more difficult [33]. In this study, the fact that 3 mm wounds on the tongue healed completely in two weeks, while palatal wounds of the same size remained almost unhealed, may be due to thermal osteonecrosis. In other words, burns on the palate, regardless of size, may affect the underlying bone, making it difficult to assess wound healing with new treatment regimens.

Diabetes increases the risk of wound infection and delays wound healing [34]. Oral ulcers in diabetic patients can experience delayed healing and an increased risk of infection, even if diabetes is well-controlled, necessitating additional treatment measures such as antibiotics or antimicrobial mouthwashes [11, 12]. In a study where an 8 mm diameter oral wound was created in the buccal mucosa of rats, it was observed that diabetic rats took 15 days to heal, whereas normal rats healed after 10 days [15]. Similarly, in another study where a 1.0 by 1.0 by 1.5 mm ulcer was created on the tongue of rats using dental rongeurs, normal rats showed complete epithelialization after 7 days, while diabetic rats still had inflamed wounds by the 14th day [20]. In this study as well, only the group with 3 mm burn wounds on the tongue was completely healed after two weeks, while the other groups showed delayed healing.

The findings from this study suggest that diabetic rats with oral burn wounds, particularly those with larger ulcers on the tongue, represent a suitable model for investigating the healing efficacy of new treatments for oral ulcers. However, palate burn wounds are less reliable due to the high variability in healing outcomes and the technical sensitivity involved in their induction. Additionally, the healing process in tongue burn wounds is more consistent and less variable. For experiments with a 1-week duration, using the tongue model, regardless of ulcer size, is advisable, as neither size typically achieves complete healing within this timeframe. For a 2-week experimental period, the 5 mm tongue model is particularly useful, as it shows a slower healing progression compared to the 3 mm tongue model, allowing for a more detailed evaluation of the therapeutic effects of potential treatments. Future research should focus on evaluating potential therapeutic agents using this model to develop effective treatment strategies for oral ulcer management.

This study has several limitations. First, this study was limited in scope to the analysis of TNF-α and IL-1β as markers of the inflammatory response in diabetic wound healing. Although these cytokines are critical to understanding the inflammatory phase, future studies using this model should consider evaluating a broader range of factors such as EGF, IGF, TGF-β, PDGF, and NGF, which contribute to the regeneration of oral mucosal tissues. Additionally, due to the lack of bone specimen collection and histological analysis, this study could not confirm osteonecrosis in the palatal bone.

## Conclusion

Within the limitations of the study, a 3 mm or 5 mm burn wound on the tongue of diabetic rats can serve as a useful animal model for assessing new wound healing treatments, especially within the first week. However, for research extending to the second week, the 5 mm burn wound model on the tongue might be more advantageous.

## Supplementary Information

Supplementary Material 1.

Supplementary Material 2.

## Data Availability

All data were shown in this manuscript.

## Abbreviations

STZ: Streptozotocin
H&E: Hematoxylin and eosin
TNF-α: Tumor necrosis factor-alpha
IL-1β: Interleukin-1 beta
PBS: Phosphate-buffered saline
