# Supplementary figures and images for: Establishment of an oral burn model in streptozotocin-induced diabetic rats
Source: Maxillofac Plast Reconstr Surg. 2024 Dec 30;46(1):43. doi: 10.1186/s40902-024-00453-6 (PMC11685340; doi:10.1186/s40902-024-00453-6)

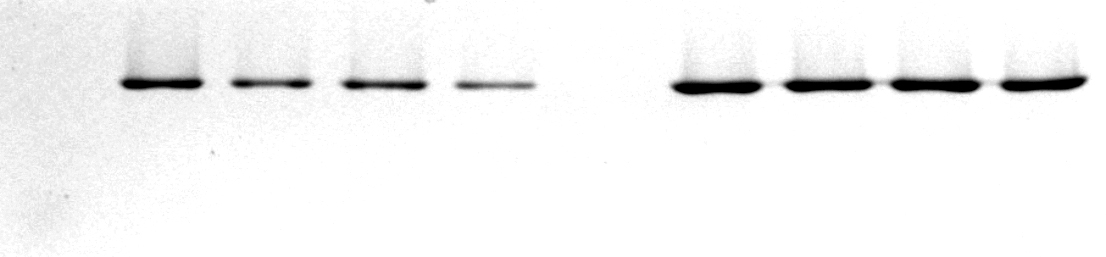

Supplement: Supplementary file 1 — Supplementary Material 1. [file 40902_2024_453_MOESM1_ESM.zip › IL-1b -1.jpg]

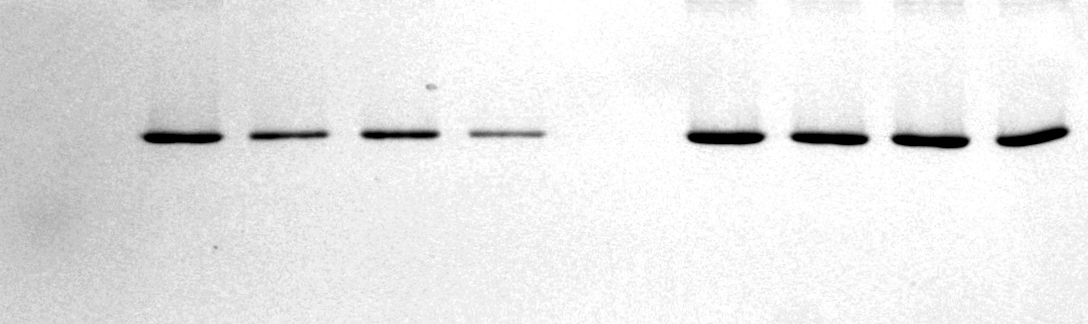

Supplement: Supplementary file 1 — Supplementary Material 1. [file 40902_2024_453_MOESM1_ESM.zip › IL-1b -2.jpg]

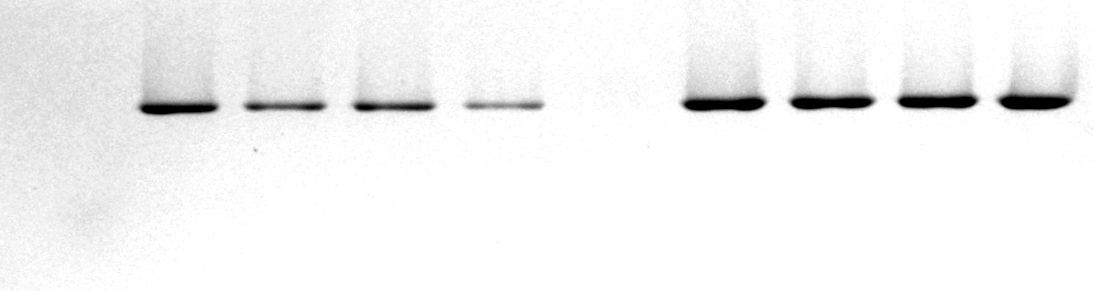

Supplement: Supplementary file 1 — Supplementary Material 1. [file 40902_2024_453_MOESM1_ESM.zip › IL-1b -3.jpg]

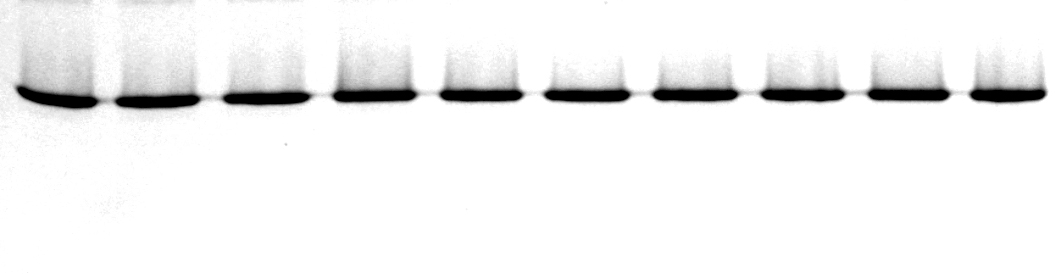

Supplement: Supplementary file 1 — Supplementary Material 1. [file 40902_2024_453_MOESM1_ESM.zip › IL-1b -actino.jpg]

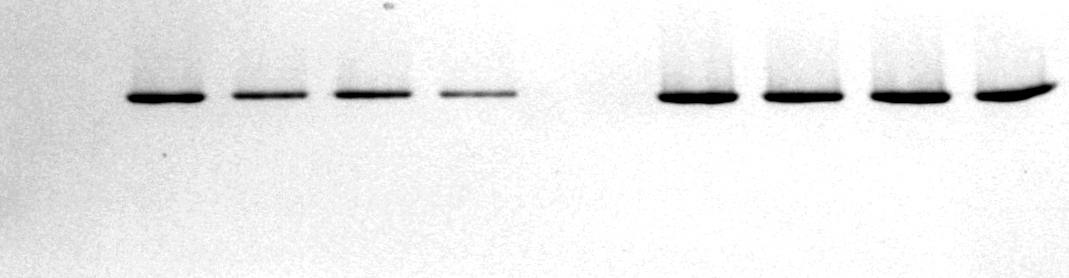

Supplement: Supplementary file 1 — Supplementary Material 1. [file 40902_2024_453_MOESM1_ESM.zip › TNF-a -1.jpg]

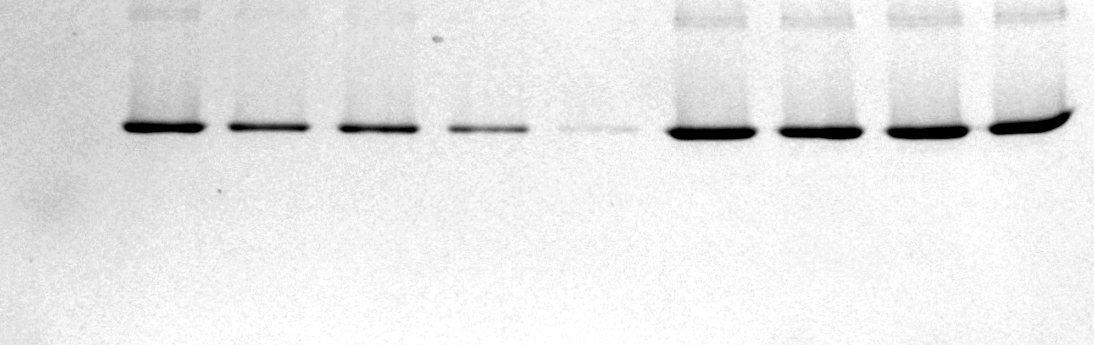

Supplement: Supplementary file 1 — Supplementary Material 1. [file 40902_2024_453_MOESM1_ESM.zip › TNF-a -2.jpg]

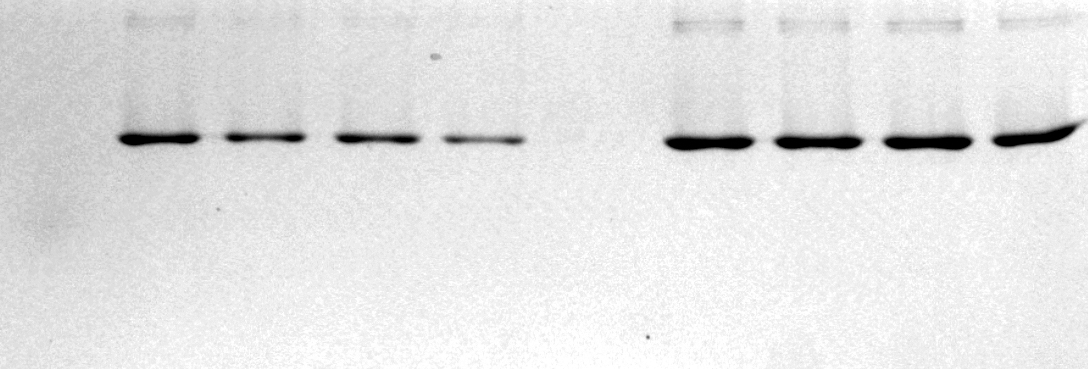

Supplement: Supplementary file 1 — Supplementary Material 1. [file 40902_2024_453_MOESM1_ESM.zip › TNF-a -3.jpg]

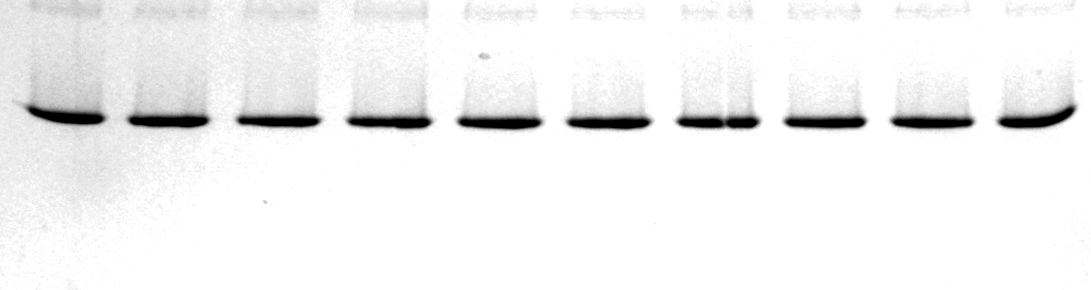

Supplement: Supplementary file 1 — Supplementary Material 1. [file 40902_2024_453_MOESM1_ESM.zip › TNF-a -actino.jpg]
